# Development of an Integrated Continuous Manufacturing Process for the rVSV-Vectored SARS-CoV-2 Candidate Vaccine

**DOI:** 10.3390/vaccines11040841

**Published:** 2023-04-14

**Authors:** Zeyu Yang, Barbara Cristina Martins Fernandes Paes, Julia Puppin Chaves Fulber, Michelle Yen Tran, Omar Farnós, Amine A. Kamen

**Affiliations:** Viral Vectors and Vaccines Bioprocessing Group, Department of Bioengineering, McGill University, Montreal, QC H3A 0G4, Canada

**Keywords:** COVID-19, SARS-CoV-2, viral vectored vaccine, recombinant vesicular stomatitis virus, perfusion, upstream process, downstream process, continuous manufacturing

## Abstract

The administration of viral vectored vaccines remains one of the most effective ways to respond to the ongoing novel coronavirus disease 2019 (COVID-19) pandemic. However, pre-existing immunity to the viral vector hinders its potency, resulting in a limited choice of viral vectors. Moreover, the basic batch mode of manufacturing vectored vaccines does not allow one to cost-effectively meet the global demand for billions of doses per year. To date, the exposure of humans to VSV infection has been limited. Therefore, a recombinant vesicular stomatitis virus (rVSV), which expresses the spike protein of SARS-CoV-2, was selected as the vector. To determine the operating upstream process conditions for the most effective production of an rVSV-SARS-CoV-2 candidate vaccine, a set of critical process parameters was evaluated in an Ambr 250 modular system, whereas in the downstream process, a streamlined process that included DNase treatment, clarification, and a membrane-based anion exchange chromatography was developed. The design of the experiment was performed with the aim to obtain the optimal conditions for the chromatography step. Additionally, a continuous mode manufacturing process integrating upstream and downstream steps was evaluated. rVSV-SARS-CoV-2 was continuously harvested from the perfusion bioreactor and purified by membrane chromatography in three columns that were operated sequentially under a counter-current mode. Compared with the batch mode, the continuous mode of operation had a 2.55-fold increase in space–time yield and a reduction in the processing time by half. The integrated continuous manufacturing process provides a reference for the efficient production of other viral vectored vaccines.

## 1. Introduction

The novel coronavirus disease 2019 (COVID-19), i.e., the severe acute respiratory syndrome coronavirus 2 (SARS-CoV-2), has resulted in the infection of 754.0 million people, including 6.8 million fatalities, since the first patient was reported in late 2019. Vaccination has been proven to be the best method by which to deal with the pandemic [1]. In the global vaccine campaign, mRNA vaccines from Pfizer-BioNTech and Moderna, the protein subunit vaccine from Novavax, and viral vectored vaccines from Janssen and AstraZeneca have played an important role in combating the COVID-19 pandemic. Among them, viral vectored vaccines represent one of the latest strategies in vaccine development. However, the efficiency of using viral vectored vaccines in a clinic setting can be hindered by the pre-exposure of the population to the vectors [2]. To overcome this issue, Janssen developed Ad26.COV2-S using a rare human adenoviral vector Ad26, while AstraZeneca selected the chimpanzee adenoviral vector ChAdY25 to produce ChAdOX1-nCoV [3,4]. Thus, selecting a viral vector with limited exposure to human populations is of great importance in the development of viral vectored vaccines [5,6,7].

As of February 2023, the WHO reports that a total of 13.3 billion vaccine doses have been administered. To meet the global demand of billions of doses annually, intensifying the production process is an endless task for manufacturing companies. Compared with batch-based processing, continuous processing—which is already established in many other manufacturing fields such as the chemical and food industries—is a promising method by which to achieve this goal, and it is rapidly gaining adherence by several leading biopharmaceutical companies [8,9,10]. Sanofi and Genzyme, for instance, have developed perfusion platforms to realize continuous processing [11]. However, most of the process intensification cases were limited within upstream or downstream processes only and were focused on the production of monoclonal antibodies, thus resulting in less efficiency and product diversity [12,13,14]. It is of great urgency to develop a continuous manufacturing mode integrating both upstream and downstream processes to produce viral vectored vaccines.

In this study, a recombinant vesicular stomatitis virus (rVSV) was used as the vector due to the low incidence rate of humans infected by VSV. This was achieved by building on the technical and clinical advancements that led to the recent approval of the VSV-based vaccine to control the Ebola epidemic [15]. The rVSV-SARS-CoV-2 vaccine candidate was constructed as rVSV_Ind_-*msp*-S_F_-*Gtc*. It is based on a recombinant VSV_Ind_ (GML) mutant that expresses the SARS-CoV-2 spike protein gene [16]. rVSV-SARS-CoV-2 was produced using a Vero cell line, which is the most used continuous cell line in viral vectored vaccine manufacturing. To enable effective process scalability, the Vero cells have been adapted to grow in suspension cultures in a commercially available serum-free medium. In addition, they have been implemented in the stirred-tank bioreactor production of NDV and VSV [17,18,19,20]. Since rVSV-SARS-CoV-2 is a temperature-sensitive construct, the optimal operation temperature was evaluated in a modular bioreactor Ambr 250 unit. Combined with the previously optimized multiplicity of infection (MOI), the cell density at infection, media formulation, and the conditions for producing rVSV-SARS-CoV-2 in an upstream process were determined [19]. Furthermore, a streamlined downstream process was developed for purifying rVSV-SARS-CoV-2. The viral materials were first treated by Benzonase and then subjected to clarification in order to reduce the double-strand DNA (dsDNA) and cell debris. After a clarification step, the supernatant was purified by an anion exchange membrane. In this step, citrate was used as an interferent agent to compete with proteins, thus increasing the purity of rVSV-SARS-CoV-2 and thereby avoiding additional polishing steps. The design of experiment (DOE) was performed to determine the optimal anion exchange membranes, pH values, and salts.

After identifying the optimal conditions for production and the purification of the rVSV-SARS-CoV-2 vectored vaccine, an end-to-end continuous manufacturing process was developed by integrating the perfusion mode and three-column periodic counter-current chromatography strategy. In an upstream process, the space–time yield of the rVSV-SARS-CoV-2 produced in a perfusion bioreactor was 2.55-fold higher than those produced in a batch bioreactor. In a downstream process, since the loading and elution steps are performed simultaneously, the continuous downstream process cut the processing time in half. Such integrated continuous manufacturing processes provide many advantages. First, continuous processing requires smaller quantities of buffers and cell media, less containers, and reduced footprint, thus leading to a lower cost. Second, product quality is enhanced since the nutrient is constantly supplemented in the bioreactor and the sensitive bioproducts are continuously separated and purified. Further, a closed-loop production in a continuous mode helps eliminate major causes of contamination. The integrated continuous manufacturing process not only improves efficiency and avoids contamination, but also creates a generic platform for the continuous production of viral vectored vaccines.

## 2. Materials and Methods

### 2.1. Cell Line and Virus

The suspension Vero cell line was provided by National Research Council Canada (NRC) [17]. It was further adapted to grow in suspension, a serum-free medium, and an MDXK medium (Xell AG) [18]. The adaptation method to suspension cultures in serum-free media has been described previously. Regarding cell passage frequency, when the Vero cells entered the late exponential phase, the cell culture was transferred into 50 mL centrifugal tubes and then centrifuged for 5 min at 800× *g*. After discarding the supernatant, the cell pellet was resuspended in a fresh MDXK medium supplemented with 4 mM of GlutaMax to a cell density of 0.5–0.8 × 10^6^ cells/mL in 250 mL shake flasks, which were operated at a 50 mL working volume. The Vero cells were incubated in a shaker at 37 °C, 135 rpm and 5% CO_2_ for 3 days.

The rVSV-vectored COVID-19 vaccine candidate rVSV_Ind_-*msp*-S_F_-*Gtc* was designed to express the full-length spike protein gene of SARS-CoV-2 (GenBank No: JX869059.2). The construction details have been described previously [16]. The infectious and genomic viral titer for the rVSV_Ind_-*msp*-S_F_-*Gtc* viral seed stock was 1.48 × 10^9^ TCID_50_/mL and 8.86 × 10^9^ VG/mL, respectively.

### 2.2. Cell Growth and rVSV-SARS-CoV-2 Production

#### 2.2.1. Ambr250 Modular Bioreactor Virus Studies

The operation conditions for the production of rVSV-SARS-CoV-2 were determined via an Ambr 250 modular system (Sartorius Stedim, Göttingen, Germany). For the Ambr 250 modular system runs, single-use 250 mL baffled vessels with two three-pitched-blade impellers (∅26 mm), as well as sensors for dissolved oxygen (DO) concentration and pH probes were used. The following parameters were controlled in the system: pH at 7.2 (dead band: 0.02), temperature at 37 °C, DO at 50%, and stirring at 220 rpm. The DO concentration was maintained by pure oxygen through a sparger when necessary, and an additional minimum sparged air flow of 0.05, or 0.1 mL/min, to prevent culture flowing back up the sparger when not in use. An air flow in the headspace of 0.1 or 1 mL/min was kept at a constant value. The pH was regulated by the addition of CO_2_ in the headspace, or via the injection of NaHCO_3_ (90 g/L). Around 1 × 10^6^ cells/mL were seeded in 220 mL of an MDXK medium supplemented with 4 mM of _L-_Glutamine. The cells were infected at a MOI of 0.01. Temperatures of 31 °C and 34 °C during the production phase of rVSV-SARS-CoV-2 were assessed. The supernatant was collected every 12 h until 60 h post infection (hpi) for further quantitative analyses.

#### 2.2.2. rVSV-SARS-CoV-2 Production in a Batch Bioreactor

The productions of rVSV-SARS-CoV-2 were performed in a 3 L Applikon bioreactor unit using the Applikon software (Applikon Biotechnology, Delft, Netherlands) for data acquisition and control. The bioreactor was equipped with a dissolved oxygen probe (Cole-Parmer, Vernon Hills, IL, USA), a pH probe (Cole-Parmer, Vernon Hills, IL, USA), a temperature probe (Cole-Parmer, Vernon Hills, IL, USA), and a capacitance probe (Aber Instruments, Aberystwyth, UK). The O_2_ and air/CO_2_ gas flow rates were controlled by the bioreactor unit. The suspension Vero cells seed cultures were grown in polycarbonate shake flasks (Corning, Somerville, MA, USA). After harvesting, the Vero cells were resuspended in a fresh medium for the purpose of inoculation. The bioreactor was inoculated on day 0 at a cell density of 0.30 × 10^6^ cells/mL in a 2 L working volume. The cell culture was agitated at 120 rpm and maintained at 37 °C. The pH was set at 7.2 and controlled by adding 90 g/L NaHCO_3_ or by injecting CO_2_ in the headspace of the bioreactor. The DO concentration was kept at a 50% air saturation and regulated by injecting O_2_ through a sparger if necessary. The cells were infected by rVSV-SARS-CoV-2 at 0.01 MOI, and the temperature was lowered to 31 °C until the end of the run [19,20].

#### 2.2.3. rVSV-SARS-CoV-2 Production in a Perfusion Bioreactor

The perfusion bioreactor culture was used in a 3 L Applikon bioreactor system (Applikon Biotechnology, Delft, Netherlands), equipped as previously described, with a DO probe, pH probe, temperature probe, and capacitance probe. Nova Flex2 (Nova Biomedical, Waltham, MA, USA) was used for the metabolic analysis. The flow rate of O_2_ and air/CO_2_ was controlled and monitored by the bioreactor system. An MDXK medium supplemented with 4 mM of _L-_glutamine (GE Healthcare, Chicago, IL, USA) was stored in a feeding bottle at 4 °C. The viral product released in the supernatant was continuously harvested from the perfusion bioreactor and stored at 4 °C. The perfusion system was based on acoustic cell retention technology. The cell retention system is an acoustic filter that is controlled by a BioSep controller (ADI 1015) (Applikon Biotechnology, Delft, Netherlands), the details of which have been described previously [17]. In brief, the acoustic filter is a cell retention device that is reliable during the perfusion of mammalian cell cultures. Key operating parameters—including perfusion and recirculation flow rates—acoustic power, and backflush frequency are determined according to the separation efficiency and to the retention of cell viability.

The bioreactor was inoculated on day 0 at a cell density of 0.32 × 10^6^ cells/mL in a 2 L working volume. The cell culture was agitated at 120 rpm and maintained at 37 °C. The pH was set at 7.2 and controlled by a NaHCO_3_ solution and CO_2_. The DO concentration was set to a 50% air saturation and regulated by the addition of O_2_ through the sparger. Cells first grew under the batch mode during the first 3 days. On day 3, the perfusion culture started at a 0.6 vessel volume per day (VVD). The cells were infected by rVSV-SARS-CoV-2 on day 5 at a 0.01 MOI. The temperature was set to 31 °C until the end of the run. From 0 hpi to 12 hpi, the bioreactor was operated under a batch mode to retain the viral inoculum within the bioreactor, thus maximizing the cell virus contact time. At 12 hpi, the perfusion mode was resumed with 1 VVD. The progeny viral material was collected every 12 h thereafter for downstream processing.

### 2.3. Calculation of rVSV-SARS-CoV-2 Productivity in Batch Mode and Continuous Mode

In an upstream process, there are various descriptions that are used when comparing the productivity of batch and perfusion modes; for example, yield, space–time yield (*STY*), volumetric productivity (*VP*), and cell-specific productivity (*CSP*) [21]. Yield is defined as the total accumulated infectious viral particles (IVP) since process start. For the batch mode, yield equals the infectious viral titer multiplied by the working volume of the bioreactor:

(1) $$Y_{Batch,i}=c_{P,i}*V_{Batch,i}$$

Equation (1): *Y* = yield [IVP], *c_P_* = infectious viral titer [TCID_50_/mL], *V* = working volume [mL], and *i* = day.

For the perfusion mode, the IVP in the harvest pool and the harvest flow rate need to be considered when calculating the yield:

(2) $$Y_{Perfusion,i}=\int_{i}^{0}c_{P,i}*H_{i}*V_{Perfusion}$$

Equation (2): *Y* = yield [IVP], *c_P_* = infectious viral titer [TCID_50_/mL], *H* = harvest rate [d^−1^], *V* = harvest pool volume [mL], and *i* = day.

*STY* equals the yield divided by the product volume and process duration, and it is another parameter by which to compare the productivities between batch and perfusion modes regarding the overall productivity of a process:

(3) $$STY_{i}=\frac{Y_{i}}{V_{Perfusion,Batch}*\left(t_{i}-t_{0}\right)}$$

Equation (3): *STY* = space–time yield [IVP mL^−1^ d^−1^], *Y* = yield [IVP], *t* = process time [d], *V* = volume [mL], and *i* = day.

For the batch mode, the equation can be simplified since the working volume is in a steady state:

(4) $$STY_{Batch,i}=\frac{c_{P,i}}{\left(t_{i}-t_{0}\right)}$$

Equation (4): *STY* = space–time yield [IVP mL^−1^ d^−1^], *c_P_* = infectious viral titer [TCID_50_/mL], *t* = process time [d], and *i* = day.

*VP* [IVP mL^−1^ d^−1^] refers to the current productivity at a given time. For the batch mode, *VP* can be calculated according to the following:

(5) $$VP_{Batch,i}=\frac{dc_{P}}{dt}=\frac{c_{P,i}-c_{P,i-1}}{t_{i}-t_{i-1}}$$

Equation (5): *VP* = volumetric productivity [IVP mL^−1^ d^−1^], *c_P_* = infectious viral titer [TCID_50_/mL], *t* = specific time [d], and *i* = day.

For the perfusion mode, *VP* can be simplified since viral particles are harvested in the same intervals:

(6) $$VP_{Perfusion,i}=c_{P,i}*H_{i}$$

Equation (6): *VP* = volumetric productivity [IVP mL^−1^ d^−1^], *c_P_* = infectious viral titer [TCID_50_/mL], *H* = harvest rate [d^−1^], and *i* = day.

*CSP* [IVP cell^−1^ d^−1^] is the specific productivity per cell. It can be determined in accordance with the following:

(7) $$CSP_{i}=\frac{VP_{i}}{X_{i}}$$

Equation (7): *CSP* = cell-specific productivity [IVP cell^−1^ d^−1^], *VP* = volumetric productivity [IVP mL^−1^ d^−1^], *X* = cell density [cell mL^−1^], and *i* = day.

### 2.4. Downstream Processing for rVSV-SARS-CoV-2

#### 2.4.1. Development and Optimization of the Downstream Process

##### DNase Treatment and Clarification

The downstream process includes DNase treatment, clarification, ion exchange chromatography, as well as buffer exchange and concentration. After harvesting, benzonase (Millipore Sigma, Burlington, MA, USA) was added to the shake flask at 10 units/mL; it was then incubated in a shaker at 135 rpm and kept at 37 °C for 1 h. Two steps of clarification were used to clarify the viral material. The virus was first centrifuged at 800× *g* for 5 min for cellular debris removal to reduce the bioburden. Then, the material was filtered by 0.45 μm syringe filters (Millipore Sigma, Burlington, MA, USA) [22].

##### Evaluation of the Interfering Agent in the Chromatography Step

The clarified viral material was divided into two groups and purified by Mustang Q XT Acrodisc (Pall, Port Washington, NY, USA). In the first group, the equilibration buffer contained 20 mM of Tris-HCl and 100 mM Trisodium citrate dihydrate (pH 7.4), while elution buffer A contained 20 mM of Tris-HCl (pH 7.4). In addition, elution buffer B contained 20 mM of Tris-HCl and 2 M of NaCl (pH 7.4). In the second group, the buffers were prepared without citrate.

##### The Design of Experiment (DoE) in the Chromatography Step

In the chromatographic step, the optimal purification conditions including pH, anion exchange membrane, and salts were evaluated [23]. The pH values were evaluated from pH 6.6–9.0. When the pH was 6.6, the equilibration buffer contained 20 mM of HEPES and 100 mM of Trisodium citrate dihydrate. Meanwhile, elution buffer A contained 20 mM of HEPES, and elution buffer B contained 20 mM of HEPES and 2 M of salt. When the pH was 7.4, 8.2, and 9.0, the equilibration buffer contained 20 mM of Tris-HCl and 100 mM of Trisodium citrate dihydrate. Meanwhile, elution buffer A contained 20 mM of Tris-HCl, and elution buffer B contained 20 mM of Tris-HCl and 2 M of salt. Two anion exchange membranes from different vendors were evaluated. One was a NatriFlo HD-Q Recon Mini (Millipore Sigma, Burlington, MA, USA), the other was a filter syringe Mustang Q XT Acrodisc (Pall, Port Washington, NY, USA). The different types of salts in elution buffer B were evaluated. The different formulations of elution buffer B included elution buffer B1 (20 mM of HEPES or 20 mM of Tris-HCl, as well as 2.0 M of NaCl), elution buffer B2 (20 mM of HEPES or 20 mM of Tris-HCl, as well as 2.0 M of Na_2_SO_4_), elution buffer B3 (20 mM of HEPES or 20 mM of Tris-HCl, as well as 2.0 M of Na_3_PO_4_).

##### Buffer Exchange and Concentration

The eluent was subjected to buffer exchange and concentration. The viral material was added in the 30 kDa Amicon centrifugal filter (Millipore Sigma, Burlington, MA, USA). The centrifugal filters were centrifuged at 800× *g* for 30 min or longer until the volume was less than 1 mL. Then, 10 mL of a neutralization buffer (50% *w*/*v* sucrose; 20 mM MgCl_2_; 25 mM Tris-HCl; and pH 7.4) was added into the centrifugal filter to conduct the buffer exchange. The whole buffer exchange operation was performed three times to change the buffer to the neutralization buffer. The viral material was concentrated in order to make the infectious viral titer meet the requirement for animal experiments and further stability tests. The final product was then stored at −80 °C.

#### 2.4.2. Establishment of the Continuous Downstream Process

**Figure 1:**
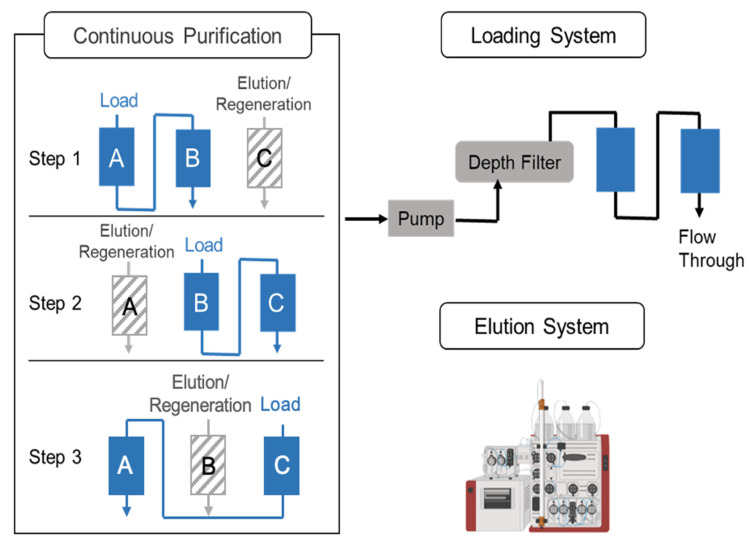
The schematic of continuous flow chromatography, including the loading system and the elution system.

The downstream process started at 24 hpi, which is when there is 1 L of viral material generated in the harvest pool. A three-column periodic counter-current chromatography strategy was implemented in the continuous downstream process. There were three Mustang Q XT Acrodisc membranes (membrane A, B, and C); two systems, including a clarification/loading system; and a wash/elution/regeneration system involved. Regarding the clarification/loading system, the viral material was pumped into the depth filter (Millipore, Sigma, Burlington, MA, USA), as well as into membrane A and membrane B, in series, which was achieved via a peristaltic pump at 2 mL/min (Figure 1). When loading 166 mL of the virus material, membrane A was transferred to the AKTA for wash, elution, and regeneration steps, while membrane C was connected to membrane B in order to be loaded. Similar sequences were operated until processing all the material from the DNase treatment [24,25]. The duration of the loading and AKTA processing were set to be consistent.

After collecting the eluent, a tangential flow filtration (TFF) step was performed to realize buffer exchange and concentration. Then, 60 mL of eluent was diluted to 300 mL with a 5% *w*/*v* sucrose solution and concentrated with a 100 kDa TFF filter (Repligen, Waltham, Massachusetts, USA). The final product was stored in a neutralization buffer (2 mM of MgCl_2_, 5% *w*/*v* sucrose, and 20 mM of Tris-HCl at pH 7.4) at –80 °C.

### 2.5. Analytical Methods and Quality Control

The Vero cell density and viability were measured via the VI-CELL XR cell counter (Beckman Coulter, Brea, CA, USA). The dsDNA was quantified using the PicoGreen^®^ dsDNA quantitation assay kit (Invitrogen, Waltham, MA, USA). The total protein in samples from each step was quantified using DC protein assay kits (Bio-Rad, Hercules, CA, USA). All the operation methods followed the manufacturer’s protocols. For infectious viral titer, the median tissue culture infectious dose (TCID_50_) assay was used. Adherent Vero cells were seeded on a 96-well plate with around 15,000 cells in each well. After 24 h, the media was replaced by 100 μL of a serial dilution of the virus. Then, 1:10 dilutions were selected for the samples from the bioreactor runs. After incubating the plate at 31 °C with 5% CO_2_ for 4 days, the cytopathic effect of the cells were analyzed by microscope. The number of wells with positive cytopathic effects was counted and used to quantify the infectious viral titers via the Spearman and Kärber algorithm [26]. For the genomic viral titer quantification, a digital droplet polymerase chain reaction (ddPCR) assay was used. The RNA of rVSV-SARS-CoV-2 was extracted via a High Pure Viral Nucleic Acid kit (Roche, Basel, Switzerland). After RNA extraction, cDNA was generated using the iScript Select cDNA synthesis kit (Bio-Rad Laboratories, Hercules, CA, USA) with random RT-PCR primers. The cDNA was diluted to a linear range of ddPCR (1:10 to 1:10,000). Then, 5 μL of cDNA solution, EvaGreen, and primers were mixed and generated into micro droplets, as per the manufacturer’s instructions. After implementing a thermocycler program, droplets were analyzed by the droplet reader. The genomic viral titer was calculated by a conversion of copies/μL of each sample [18,20,27,28].

## 3. Results

### 3.1. rVSV-SARS-CoV-2 Production in a Batch Bioreactor

#### 3.1.1. Determination of Optimal Conditions for the Production of rVSV-SARS-CoV-2 in a Mini Bioreactor

**Figure 2:**
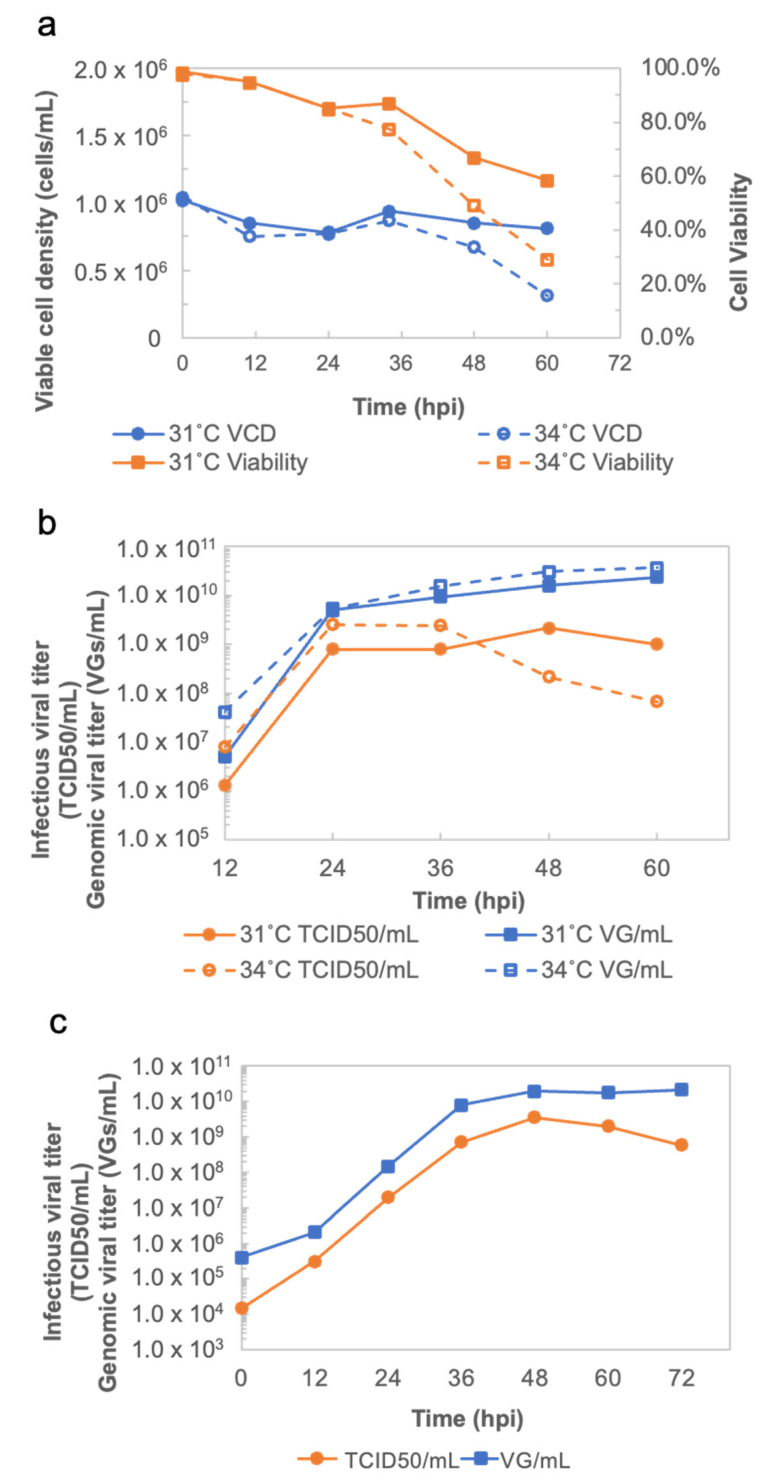
(**a**) Viable cell density and cell viability curves of rVSV-SARS-CoV-2 production at 31 °C and 34 °C in Ambr250. (**b**) Infectious viral titer and genomic viral titer curves of rVSV-SARS-CoV-2 production at 31 °C and 34 °C in Ambr250. (**c**) Infectious viral titer and genomic viral titer curves of rVSV-SARS-CoV-2 production in the batch bioreactor.

Evaluation of the optimal temperature for the production of rVSV-SARS-CoV-2 was performed using an Ambr 250 modular bioreactor unit. In the virus production phase, suspension-adapted Vero cells in two modular bioreactors were infected at 1.02 × 10^6^ cells/mL and 1.05 × 10^6^ cells/mL with an MOI of 0.01 at 31 °C and 34 °C (Figure 2a), respectively. As shown in Figure 2b, when infecting the Vero cells at 34 °C, the peak of the infectious viral titer appeared at 24 hpi with 2.54 × 10^9^ TCID_50_/mL. After 36 hpi, the functional viral particles reduced rapidly to 6.81 × 10^7^ TCID_50_/mL at 60 hpi. This confirmed that rVSV-SARS-CoV-2 is a temperature sensitive construct, which is a critical quality attribute that is conferred by design. Compared with the production at 34 °C, rVSV-SARS-CoV-2 had a stable viral production at 31 °C with a maximum of infectious viral titers of 2.15 × 10^9^ TCID_50_/mL at 48 hpi.

#### 3.1.2. Implementation of rVSV-SARS-CoV-2 Production in a Batch Bioreactor

The process for the production of rVSV-SARS-CoV-2 in a batch bioreactor was described previously [19]. The Vero cells were infected at around 1.00 × 10^6^ cells/mL after being in a 4 day culture. When infecting the cells, the temperature of the bioreactor was adjusted to 31 °C. As shown in Figure 2c, the infectious viral titer reached 3.59 × 10^9^ TCID_50_/_mL_ and peaked at 48 hpi, which is consistent with the result from the Ambr250 modular bioreactors.

### 3.2. Continuous Production of rVSV-SARS-CoV-2 in a Perfusion Bioreactor

**Figure 3:**
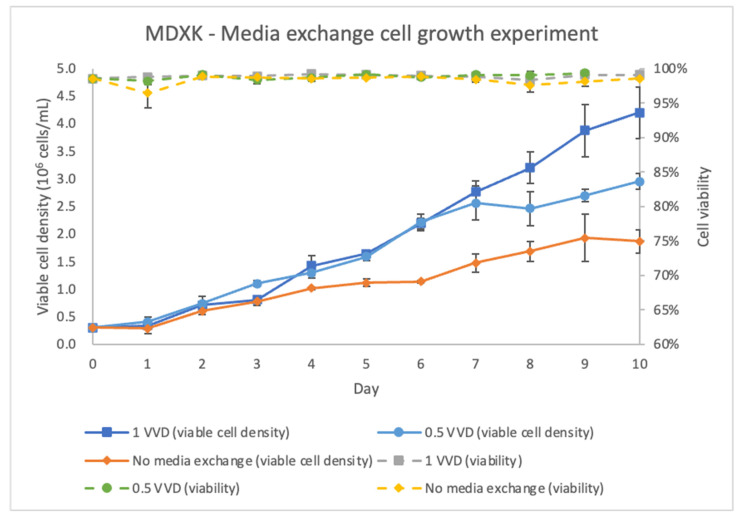
Viable cell density and cell viability in shake flasks with a media exchange at 0–1 vessel volume per day (VVD).

To determine the optimal strategy for the amplification of suspension Vero cells, cell growth studies in shake flasks were performed, in which the MDXK media were exchanged at different VVDs, ranging from 0 to 1 (Figure 3). After starting media exchange on day 3, the Vero cells that had a media exchange amplified more rapidly than those under the batch culture mode. The higher cell density resulted from the continuous nutrient supplementation and removal of metabolic waste. In the simulated perfusion mode, the cells appear under a healthier state, which probably contributed to a more effective infection process. Additionally, results showed that different VVD values did not significantly impact the viable cell density until day 7. Since the Vero cells would be infected on day 5 in the perfusion bioreactor, the moderate VVD value was selected to minimize medium consumption.

**Figure 4:**
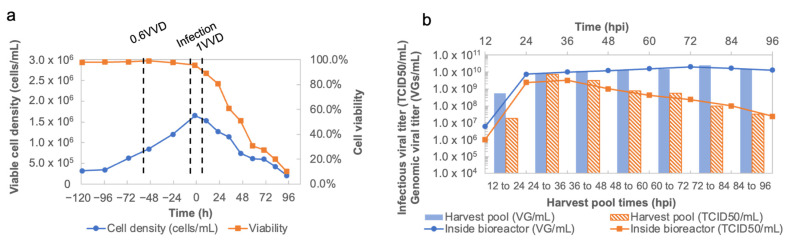
(**a**) Viable cell density and cell viability curves in cell growth and rVSV-SARS-CoV-2 production in the perfusion bioreactor. (**b**) Infectious viral titer and genomic viral titer of rVSV-SARS-CoV-2 production in the perfusion bioreactor and the harvest pool.

To operate a continuous mode of rVSV-SARS-CoV-2 production, the perfusion mode was performed in both cell growth and virus production phases. As shown in Figure 4a, the perfusion culture was initiated on day 3 post inoculation at 0.6 VVD. After 48 h, the Vero cells were infected at 1.65 × 10^6^ cells/mL using an MOI of 0.01. The perfusion mode was then switched to a batch mode to retain the virus in the bioreactor and to maximize the contact time cell/virus for infection. The temperature was kept at 31 °C throughout the run. At 12 hpi, the perfusion mode was restarted at 1 VVD to harvest the virus continuously with an acoustic filter until the end of the run. The viral materials in the harvest pool were collected each 12 h as the initial material for the downstream process. Time course curves for pH, temperature, dissolved oxygen, and capacitance are shown in Figures S1 and S2. Meanwhile, samples from the bioreactor and the harvest pool were taken in the same intervals to quantify the genomic and infectious viral productions. As shown in Figure 4b, the infectious viral titer of rVSV-SARS-CoV-2 in the bioreactor peaked at 36 hpi with 3.16 × 10^9^ TCID_50_/mL, while at 24–36 hpi, the maximal infectious viral titer reached 7.50 × 10^9^ TCID_50_/mL in the harvest pool.

### 3.3. Development and Optimization of Downstream Processing for rVSV-SARS-CoV-2

#### 3.3.1. Design of a Streamlined Downstream Process

Downstream processing for rVSV-SARS-CoV-2 is started with a clarification step, followed by DNase treatment in which the viral material was centrifuged and filtered by a 0.45 μm membrane. For the DNase treatment, benzonase was added to the harvested viral material in order to reduce the dsDNA burden. Thereafter, the supernatant was subjected to a one-step anion exchange chromatography by a membrane-based absorber. The chromatography step plays an important role in obtaining a high purity and high yield of rVSV-SARS-CoV-2 in the downstream process. In this step, the interfering agent was added into the mobile phase for improving protein removal. To test the performance of the interfering agent, the infectious viral recovery and protein concentration of the viral material that was collected from the clarification step was defined as 100%. After the implementation of the chromatographic step, it was observed that 0.28 ± 0.02% of proteins remained in the viral material. Meanwhile, when using the mobile phase without citrate, 5.32 ± 0.08% of proteins remained in the viral material. Regarding the infectious viral recovery, 56.25 ± 2.66% and 55.65 ± 2.81% of infectious viral titers were recovered using the mobile phase with and without citrate.

#### 3.3.2. Design of Experiment (DoE) for Optimal Purification Conditions in the Chromatography Step

**Figure 5:**
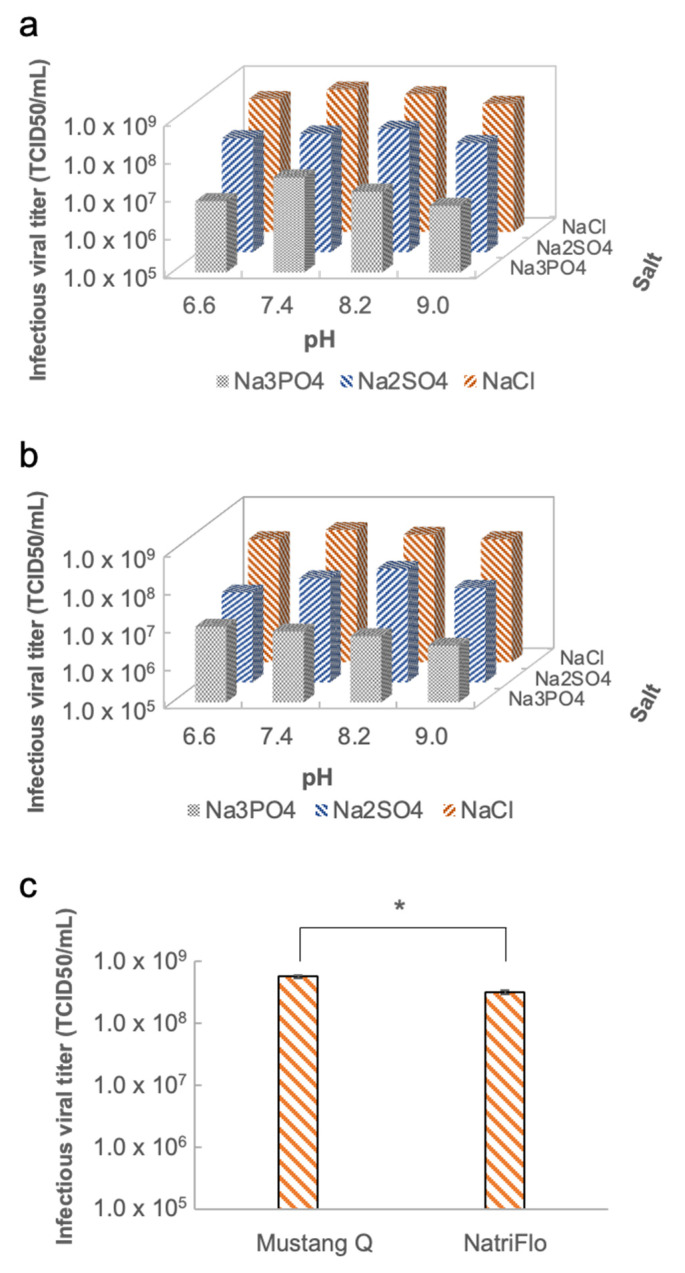
(**a**) Evaluation of the purification performance in different pH and salts with Mustang Q. (**b**) Evaluation of the purification performance in different pH and salts with NatriFlo. (**c**) Statistically significant difference between using Mustang Q and NatriFlo membranes, *p* < 0.05 (*t*-test). The asterisk denotes a statistically significance with a p-value less than 0.05.

To determine the optimal purification conditions, a two-level full factorial design of the experiment was performed (Figure 5). When assembling Mustang Q to the AKTA, results show that the salts impacted the infectious titers of rVSV-SARS-CoV-2 significantly, with decreasing trend of around one order of magnitude from NaCl to Na_3_PO_4_ (Figure 5a). Among all the combinations, the elution buffer containing NaCl at a pH of 7.4 shows the best performance for purifying rVSV-SARS-CoV-2 with the infectious viral titer of 5.62 ± 0.33 × 10^8^ TCID_50_/mL. Similarly, another anion exchange chromatographic membrane NatriFlo HD Q was selected to compare with the Mustang Q (Figure 5b). Results show that the overall infectious viral titers of rVSV-SARS-CoV-2 purified by NatriFlo HD Q were lower than those purified by Mustang Q. The infectious titer peaked at 3.16 ± 0.24 × 10^8^ TCID_50_/mL, with the best condition being a pH of 7.4 in a NaCl buffer. The DoE reveals that the NaCl buffer with pH of 7.4 when using Mustang Q is the optimal purification condition. A statistically significant difference in the infectious viral titer between using Mustang Q and NatriFlo membranes was observed, as determined by a *t*-test (*p* < 0.05) (Figure 5c).

### 3.4. Continuous Downstream Processing for rVSV-SARS-CoV-2

**Figure 6:**
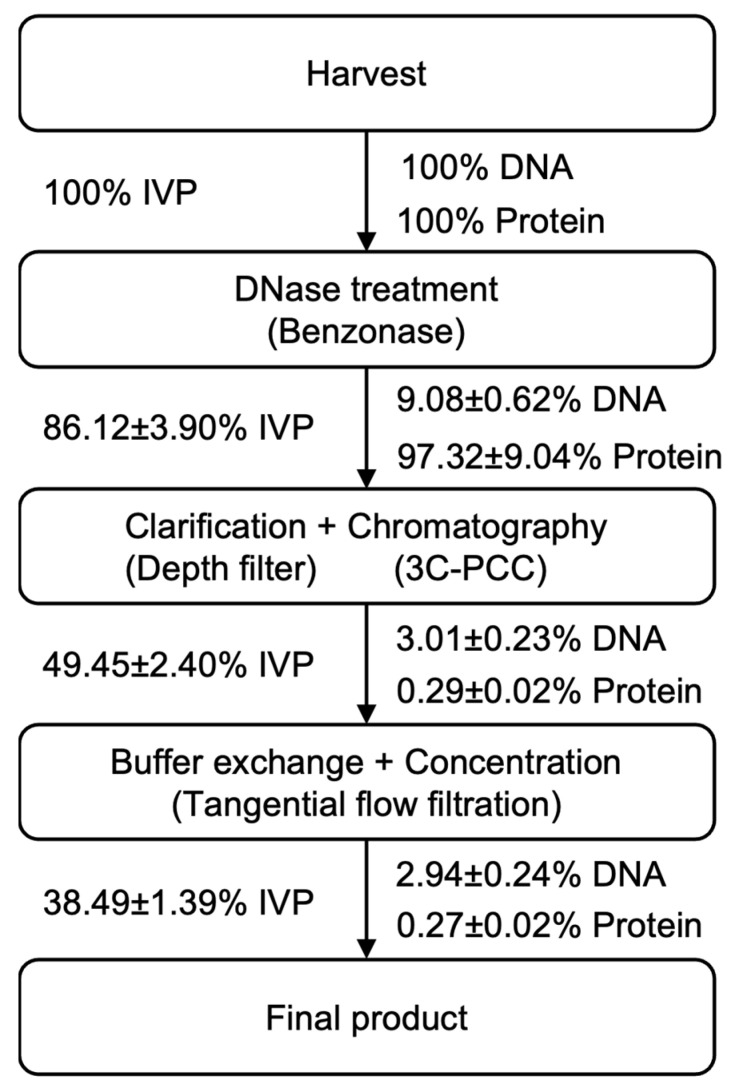
The schematic of the downstream process, infectious viral titer recovery, as well as for the DNA and protein removal.

A continuous downstream process was implemented for the efficient manufacturing of rVSV-SARS-CoV-2. Furthermore, 1 L of rVSV-SARS-CoV-2 material from the 36–48 hpi harvest pool was selected to simulate the continuous downstream process. The schematic of the process is shown in Figure 6. Samples were taken after each step of the operation unit to perform the virus titration assay and to quantify impurities, including DNA and proteins. The infectious viral recovery of viral material that was collected from the harvest pool was defined as 100%. After the DNase treatment step, around 86% of the functional viral particles were recovered and subjected to the clarification and chromatographic process. The DNA-treated viral material was kept in an ice box during this step; meanwhile, the peristaltic pump delivered viral material to the depth filter and 2 Mustang Q membranes in series. The total eluent fractions from all the circles of the wash/elution/regeneration step were quantified and mixed for the buffer exchange and the concentration step. The sum of infectious virus recoveries was 49.45% (Table 1). After using the tangential flow filter for the buffer exchange and the concentration step, the infectious viral titer of rVSV-SARS-CoV-2 was 4.05 ± 0.15 × 10^10^ TCID_50_/mL with a 38.49% infectious viral titer recovery in the final product. Regarding the purity control, more than 90% of the DNA was removed after benzonase treatment. With the contribution of the interfering agent, the proteins were significantly removed since only 0.29% of the proteins was detected after the three-column periodic counter-current chromatography step. The addition of the interfering agent avoids the additional polishing chromatography step, which contributes to the high recovery yield and the simplicity of the downstream process. Finally, the concentration of the proteins and DNA was 23.19 μg/mL and 2698.23 ng/mL, respectively. The specification of the rVSV-SARS-CoV-2 product per dose for animal tests was defined as 50 μL with 1.00 × 10^8^ PFU (Table 2). The concentration of the total proteins and DNA was at an 81.88 ng/dose and 9.52 ng/dose, respectively, which is less than the limitation of a 100 ng/dose for the host cell protein and the 10 ng/dose for DNA. Overall, the quality of the final product fulfills the primary requirement for pre-clinical studies.

### 3.5. Continuous Mode vs. Batch Mode in the Manufacturing of rVSV-SARS-CoV-2

**Figure 7:**
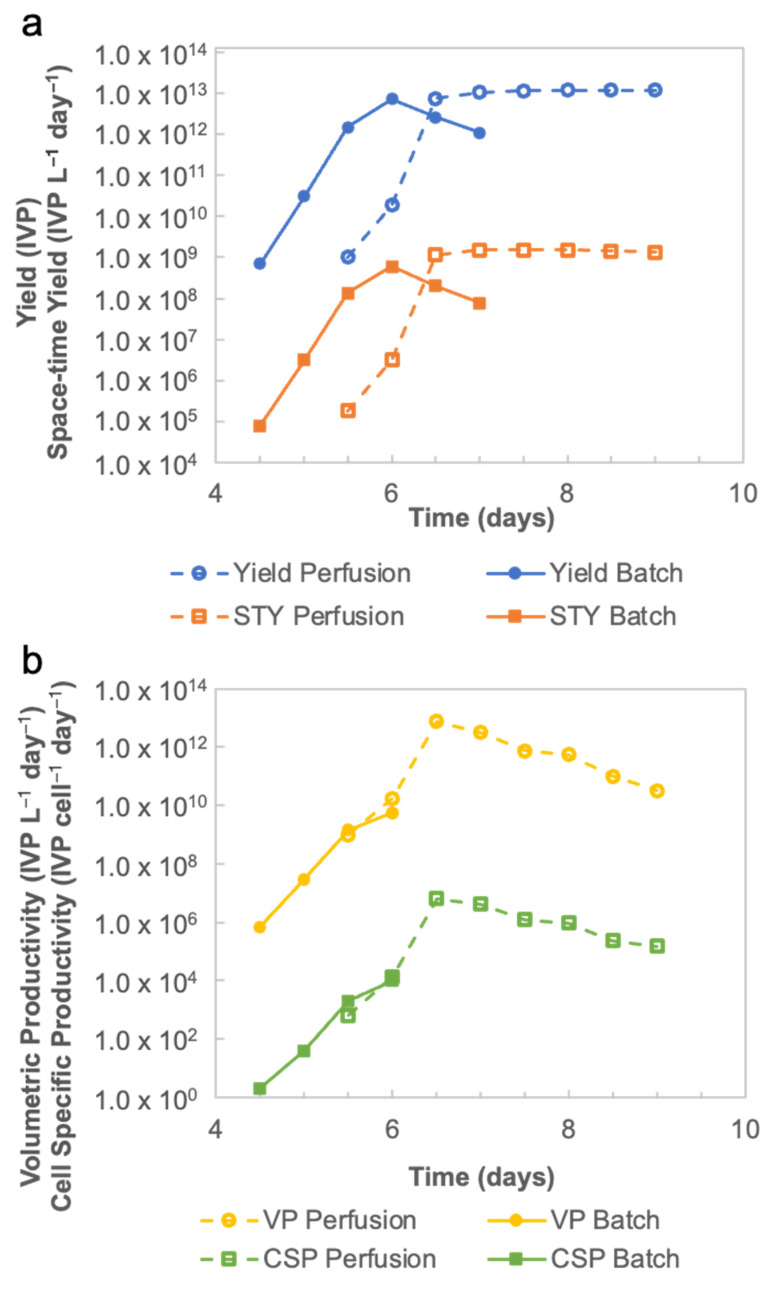
(**a**) Total yield and space–time yield curves of rVSV-SARS-CoV-2 production in the perfusion bioreactor and batch-based bioreactor. (**b**) Volumetric productivity and cell-specific productivity curves of rVSV-SARS-CoV-2 production in the perfusion mode bioreactor and the batch mode bioreactor.

According to the Equations (1)–(7), as detailed in Section 2.3, the rVSV-SARS-CoV-2 productivities under the batch and perfusion modes are shown in Figure 7. Results show a total yield of 1.09 × 10^12^ IVP for the batch mode on day 7, as well as a total yield of 1.21 × 10^13^ IVP for the perfusion mode on day 9 (Figure 7a). Considering the yield and process duration, the data suggest a maximal *STY* of 5.98 × 10^8^ [IVP mL^−1^ d^−1^] for the batch mode on day 6, and 1.53 × 10^9^ [IVP mL^−1^ d^−1^] for the perfusion mode on day 7, which represents a 2.55-fold increase. Both *VP* and *CSP* in the batch mode peaked on day 6 with 5.70 × 10^9^ [IVP mL^−1^ d^−1^] and 1.03 × 10^4^ [IVP cell^−1^ d^−1^], respectively. Meanwhile, *VP* and *CSP* for the perfusion mode peaked on day 6.5 with 3.16 × 10^12^ [IVP mL^−1^ d^−1^] and 4.27 × 10^6^ [IVP cell^−1^ d^−1^] (Figure 7b).

**Figure 8:**
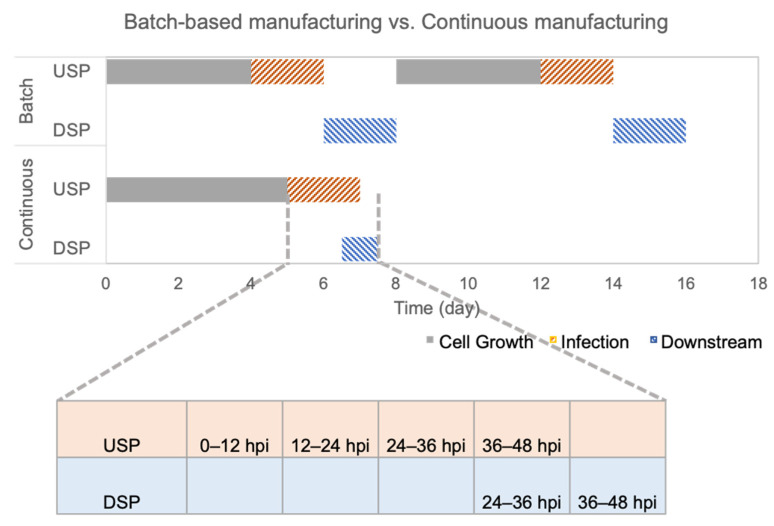
Comparison of the processing time of batch-based manufacturing and continuous manufacturing in the production of rVSV-SARS-CoV-2 in cell cultures, infection phases, and downstream processing.

For the batch mode, since the virus production peaked at 48 hpi, the upstream processing time was 6 d. The determination of the processing time for the perfusion mode was more complex. In the perfusion mode, the longer the virus production phase is maintained, the higher yield is expected to be harvested. However, a parameter for quality control, i.e., the ratio of genomic viral titer versus infectious viral titer, limits the duration of the perfusion mode as the high total particles content per dose might contribute to cytotoxicity, thus leading to cytokine storm. The ratio in the perfusion mode was 3.6 at 48 hpi, which is comparable with the ratio of 6.0 in the batch mode when harvesting. Additionally, the *STY* for the perfusion mode peaks at 48 hpi with 5.98 × 10^8^ [IVP ml^−1^ d^−1^]. Thus, the perfusion run was considered to have ended on day 7. The continuous downstream process in a perfusion bioreactor can be initiated on day 6.5 (at 36 hpi) to process the 24–36 hpi harvest pool. Another 12 h is required for processing the 36–48 hpi harvest pool. Therefore, the total processing time for the continuous mode is 7.5 d. The batch-based downstream process in a batch bioreactor starts on day 6 after harvesting. The processing time doubles because loading and elution steps happen in sequence. The total processing time for one batch bioreactor and one batch downstream process is 8 days. However, two batches and a total of 16 days are needed to reach the same yield level (10^13^ IVP), which is delivered by the continuous manufacturing mode over 7.5 days (Figure 8).

## 4. Discussion

Vero cells that grow adherently are widely used as a continuous cell line in the manufacturing of many viral vaccines, such as Ebola, influenza, rabies vaccines, etc. The Vero cell line has been proven to be a broadly susceptible cell line to viral infection. Very extensive knowledge and experience have been accumulated over many years using this cell line as a substrate for cell-culture-produced vaccines, which have been licensed worldwide [28,29,30]. 

Since Vero cells grown in suspension cultures provide significant advantages as an industrial manufacturing platform, studies have reported the successful adaptation of adherent Vero cells to grow in suspension cultures with proprietary media [17]. Further, Sascha et al. adapted the suspension Vero cells to a commercial MDXK media and optimized cell growth and virus production in shake flasks [18]; this further contributed to the demonstration of the feasibility and robustness of the approach in stirred-tank bioreactors. 

The productivity of rVSV-SARS-CoV-2 at 31 °C and 34 °C was evaluated in Ambr250. The rVSV-SARS-CoV-2 curves (Figure 2b) not only show the best time to harvest, but also demonstrate the enhanced stability of rVSV-SARS-CoV-2 at 31 °C, which makes possible to harvest the viral material continuously when implementing rVSV-SARS-CoV-2 production in a perfusion bioreactor on a larger scale. Additionally, the cell culture performances and viral production obtained in the mini bioreactors in terms of cell growth, viability, metabolism, and titer are more comparable to large scale bioreactor results. Compared to shake flasks, the mini bioreactor experiments can significantly reduce the process transfer timelines when scaling up.

After demonstrating the scalability of rVSV-SARS-CoV-2 production in 3 L bioreactors, a perfusion upstream process was implemented at 12 hpi to harvest the virus continuously. Compared with batch processing, the continuous mode of operation provides many benefits. First, the quality of the product is improved as the virus constantly flows from the bioreactor to the harvest pool, thus reducing the viral product inactivation and the contamination of the viral product by the host cell material released in the supernatant. Additionally, the scalability of the continuous mode of operation is simpler since the continuous process is primarily governed by duration instead of volumes, thus contributing to a significant reduction in capital expenditures (CAPEX), as well as making it more amenable to technology transfer, which is contrary to the perceived complexity of the operations. At the production scale, batch processing tends to require large bioreactor vessels, whereas continuous processing relies on continuously feeding media and separating virus material with a streamlined transfer to specialized chromatography processes. This leads to a reduction in energy consumption, operator time, and the costs in equipment, as is shown below.

Membrane-based ion exchange chromatography absorbers were used in this study. Compared with the packed bed-based column, the chromatographic membranes allow higher flow rates and dynamic binding capacities, which supports easier scalability. Additionally, an interference chromatographic technic was included in this step by adding an interfering agent and citrate into the supernatant and mobile phase in order to modify the molecular interactions between the viral material and the chromatographic matrix. As an interfering agent, citrate competes with other impurities with similar charges and binds with the chromatographic media, thus inhibiting the binding of molecules with weakly negative charges (e.g., proteins), such that the purity is improved [31]. When measuring the residual protein content after the chromatography step, we observed a statistically significant difference between the mobile phases with and without citrate, as determined by a *t*-test (*p* < 0.05). However, there was no significant difference in the recovery of the infectious virus. This indicates that citrate contributes to the removal of proteins without affecting the viral titer. To accelerate the optimization for operation conditions, a two-level full factorial design of the experiment was performed in which the optimal salts and pH were determined. The eluent fraction was finally subjected to buffer exchange and concentration by a centrifugal filter.

To realize a continuous mode in the downstream process, a three-column periodic counter-current chromatography-based continuous flow chromatography strategy was developed, thereby leading to a high efficiency with respect to time, buffer consumption, and the stationary phase, which was achieved by operating several membranes simultaneously. In the loading system, two membranes that are connected in series allow the chromatography step to be operated with less stringent margins. This is because when the first membrane reaches its upper limit of capacity, the unbound product can be easily collected by the second one, thus realizing an overload mode. Additionally, the clarification step is integrated into the loading system, which further contributes to an efficient process. In conjunction with the wash/elution/regeneration system, the continuous downstream process was particularly attractive for the rapid purification of temperature sensitive rVSV-SARS-CoV-2.

In this study, upstream and downstream processes are integrated to realize an end-to-end continuous manufacture for rVSV-SARS-CoV-2. A comparison of batch-based and continuous manufacturing processes was made to evaluate the efficiency from the perspective of different dimensions, including productivity and total processing time. Regarding productivity, all the parameters including yield, *STY*, *VP*, and *CSP* indicated that rVSV-SARS-CoV-2 production under the perfusion mode has a higher productivity. Regarding the processing time, the continuous mode reduced the processing time from 16 days to 7.5 days. The processing time for batch manufacturing in this paper does not even include the turn-around time between the two batches. The high productivity and low total processing times reveal that the continuous manufacturing mode possesses significant advantages.

## 5. Conclusions

In this study, we established an integrated continuous manufacturing process mode for the production of the rVSV-based SARS-CoV-2 candidate vaccine. rVSV is a promising viral vector that has limited pre-exposure to humans and has been approved for use against Ebola epidemics. Critical process parameters for the production and purification of rVSV-SARS-CoV-2 were optimized. In the upstream process, small scale studies for the production of rVSV-SARS-CoV-2 were performed in Ambr250 modular bioreactors using serum-free medium-adapted suspension Vero cells. To obtain purified rVSV-SARS-CoV-2, a streamlined downstream process was developed. In the chromatography step, the DOE for selecting anion exchange membranes, pH, and salts was performed in order to determine the optimal purification conditions.

After identifying the best operating conditions, a continuous manufacturing mode was developed and implemented, which integrated perfusion mode and continuous flow chromatography. In the upstream process, rVSV-SARS-CoV-2 was continuously harvested from the perfusion bioreactor. Compared with the batch bioreactor, the perfusion run is more efficient because of its 2.55-fold increase in space–time yield, and its more than two magnitude increase on volumetric productivity and cell-specific productivity. In other words, for a comparatively similar productivity, two batch mode runs would equal one perfusion run. In the downstream process, the three-column periodic counter-current chromatography-based continuous flow chromatography strategy reduced, by half, the downstream processing time. Therefore, the integrated continuous manufacturing process significantly increases the overall process efficiency, indicating that it is a promising strategy for viral vectored vaccine production.

## Figures and Tables

**Table 1:** The infectious viral particles’ recovery for each column in the continuous downstream process.

Sample	IVP Recovery
Elution A-1	3.35 ± 0.21%
Elution B-1	8.49 ± 0.42%
Elution C-1	10.51 ± 0.48%
Elution A-2	9.64 ± 0.43%
Elution B-2	8.81 ± 0.42%
Elution C-2	8.65 ± 0.43%
Total	49.45 ± 2.40%

**Table 2:** Final product attributes for the rVSV-SARS-CoV-2 preparations.

Product Information on rVSV-SARS-CoV-2		
Genomic Viral Titer	1.84 ± 0.08 × 10^11^ VG/mL	6.49 ± 0.27 × 10^8^ VG/dose
Infectious Viral Titer	4.05 ± 0.15 × 10^10^ TCID_50_/mL	1.00 ± 0.04 × 10^8^ PFU/dose
Protein	23.19 ± 1.64 μg/mL	81.88 ± 5.78 ng/dose
DNA	2698.23 ± 216.13 ng/mL	9.53 ± 0.76 ng/dose

## Data Availability

The data presented will be made available through the corresponding authors upon request.

## Supplementary Materials

The following supporting information can be downloaded at: https://www.mdpi.com/article/10.3390/vaccines11040841/s1, Figure S1: Time course curves of pH, temperature, dissolved oxygen, cumulative CO2, and cumulative O2 for the perfusion bioreactor.; Figure S2: Time course curve of capacitance for the perfusion bioreactor.; Figure S3: UV 280 nm, UV 260 nm, and the concentration of buffer B curves for the continuous flow chromatography.
