# Supplementary material for: Development of an Integrated Continuous Manufacturing Process for the rVSV-Vectored SARS-CoV-2 Candidate Vaccine
Source: Vaccines (Basel). 2023 Apr 14;11(4):841. doi: 10.3390/vaccines11040841 (PMC10143285; doi:10.3390/vaccines11040841)
Supplement: Supplementary file 1 [file vaccines-11-00841-s001.zip › vaccines-2280687-supplementary.pdf]

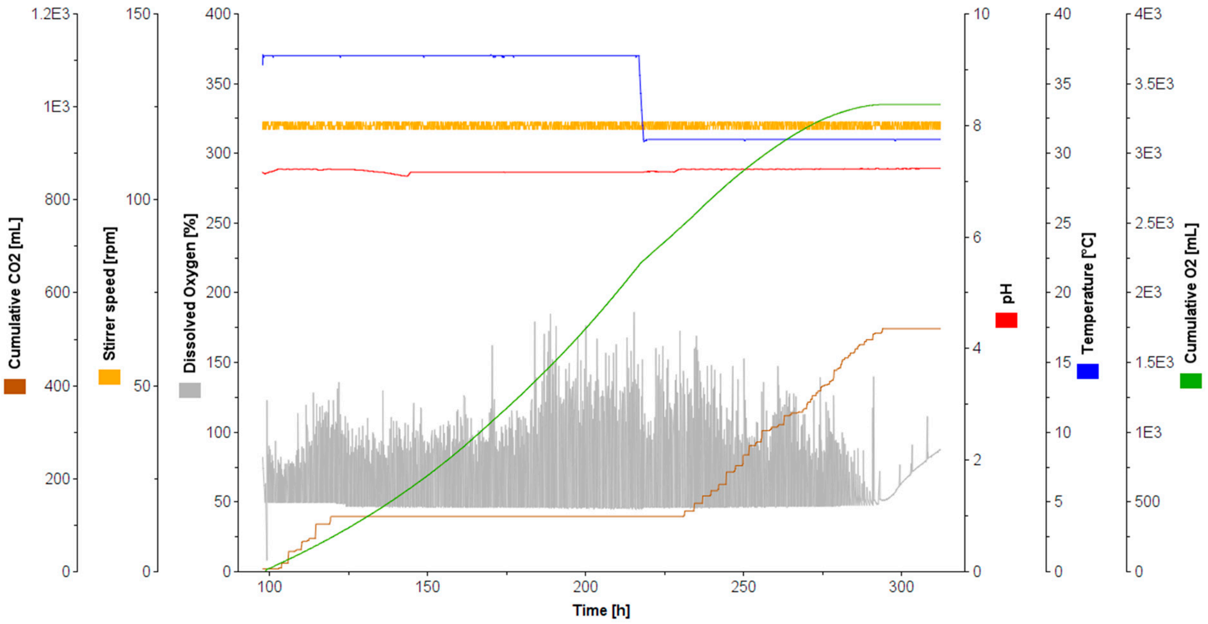

Figure S1. Time course curves of pH, temperature, dissolved oxygen, cumulative CO2, cumulative O2 for perfusion bioreactor.

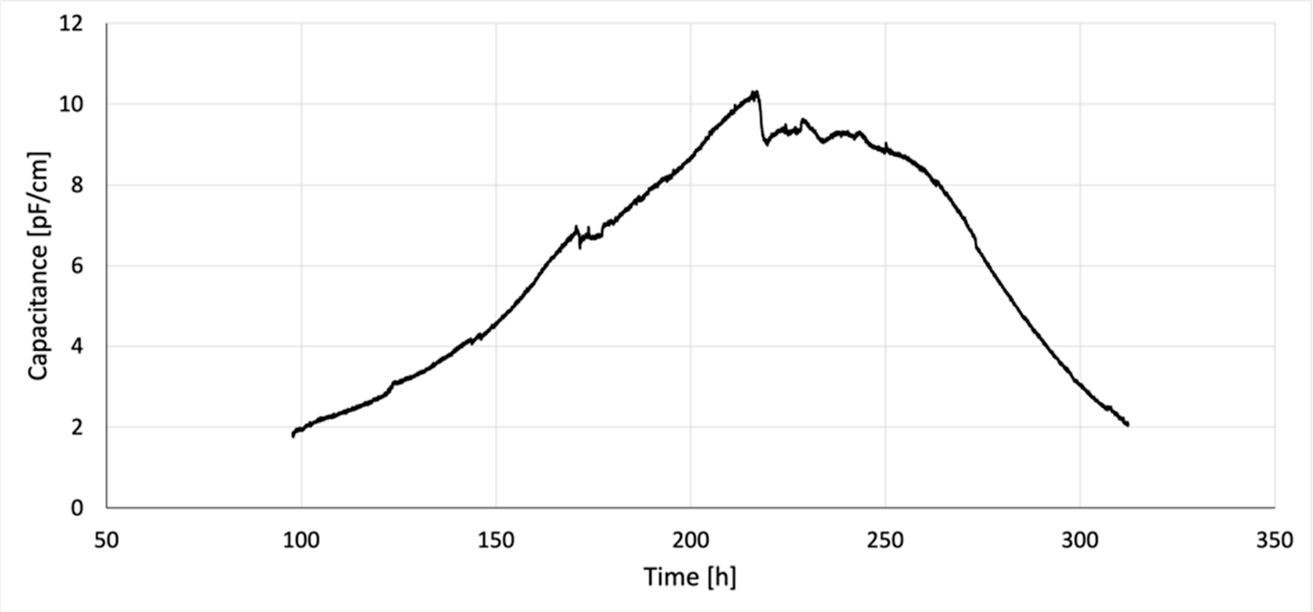

Figure S2. Time course curve of capacitance for perfusion bioreactor.

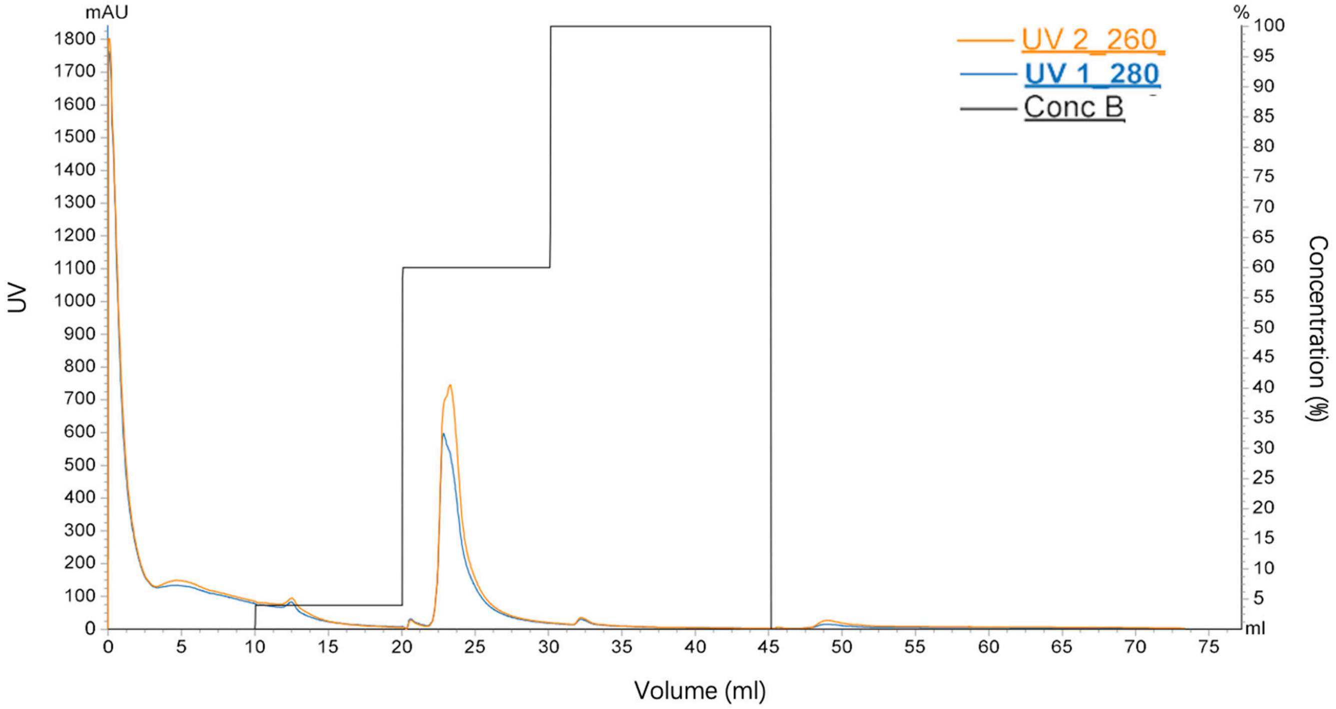

Figure S3. UV 280 nm, UV 260 nm, and concentration of Buffer B curves for continuous flow chromatography.
